# FOXO4 Inhibits the Migration and Metastasis of Colorectal Cancer by Regulating the *APC2*/β-Catenin Axis

**DOI:** 10.3389/fcell.2021.659731

**Published:** 2021-09-23

**Authors:** Yan Sun, Lin Wang, Xuehu Xu, Puqing Han, Jinghao Wu, Xuan Tian, Mingsong Li

**Affiliations:** ^1^Department of Gastroenterology, The Third Affiliated Hospital of Guangzhou Medical University, Guangzhou, China; ^2^Department of Oncology, Guangzhou Red Cross Hospital, Medical College, Jinan University, Guangzhou, China; ^3^Department of General Surgery, The Third Affiliated Hospital of Guangzhou Medical University, Guangzhou, China; ^4^Department of Gastroenterology, The Fifth Affiliated Hospital of Guangzhou Medical University, Guangzhou, China

**Keywords:** FOXO4, colorectal cancer, *APC2*/β-catenin axis, migration, metastasis

## Abstract

**Objective:** Adenomatous polyposis coli 2 (*APC2*) is a colorectal cancer (CRC) tumor-suppressor gene. The progression of several kinds of cancer is closely associated with Forkhead box O4 (FOXO4). However, the function of FOXO4 in CRC is unclear. This study focused on the role of FOXO4 and the relationship between FOXO4 and *APC2* in CRC migration and metastasis.

**Methods:** The expressions of FOXO4, *APC2*, and p(S37)-β-catenin were detected in CRC tissues by immunohistochemistry, and their correlation was analyzed using the Spearman coefficient. Chromatin immunoprecipitation was used to test whether FOXO4 binds and regulates *APC2* as a transcription factor. Either FOXO4 overexpression or APC2 knockdown was performed in CRC cell lines. The roles of FOXO4 and APC2 were investigated in CRC migration and metastasis.

**Results:** FOXO4 was downregulated in CRC tissues compared with normal tissues and positively correlated with APC2 and p(S37)-β-catenin. FOXO4 could combine the promoter region of *APC2* to upregulate its expression and increase the phosphorylated degradation of β-catenin. Stemness genes (*CD133*, *ABCG1*, and *SOX2*) were inhibited by FOXO4 overexpression in SW620 and HCT116 cell lines. Overexpressed FOXO4 suppressed epithelial–mesenchymal transition and the migration of CRC cell lines and metastasis of HCT116 in both the spleen and liver of nude mice, which was reversed by APC2 knockdown.

**Conclusion:** This research demonstrates that overexpressed FOXO4 inhibits the migration and metastasis of CRC cells by enhancing the APC2/β-catenin axis, suggesting that FOXO4 is a potential therapeutic target of CRC.

## Introduction

Colorectal cancer (CRC) is one of the most common cancer types. It has the third highest mortality rate globally (Dekker et al., 2019). With the technical progress of diagnosis and treatment, CRC mortality has been declining for many years, but the prognosis of advanced CRC is poor because of cancer metastasis (Miller et al., 2019). A considerable proportion of patients with colorectal cancer are advanced once diagnosed, especially those in high-risk groups or who do not undergo regular physical examinations. Therefore, it is crucial to explore the underlying mechanism of CRC migration and metastasis and develop treatments targeting this.

Adenomatous polyposis coli 2 (*APC2*) is the homolog gene of adenomatosis polyposis coli (APC). It plays an essential role in tumor suppression in several cancers (van Es et al., 1999; Beta et al., 2015; Ying et al., 2015). APC2 is considered as the most potential regulator for CRC deterioration. In addition, APC2 activates the Wnt/β-catenin pathway *via* participating in the formation of a multiprotein “destruction complex” (Nakagawa et al., 1998; Croy et al., 2016; Saito-Diaz et al., 2018). Abnormal activation of the Wnt/β-catenin pathway is vital for promoting epithelial–mesenchymal transition (EMT) and is widespread in many tumors, including CRC (Sebio et al., 2014).

Forkhead box O4 (FOXO4) is an influential transcriptional factor, belonging to the FOXO family, regulating multiple genes involved in apoptosis, cell cycle, cellular homeostasis, and so on (Liu et al., 2020). FOXO4 plays an essential role in transcriptional regulation in various tissues and organs, affecting the occurrence and development of many diseases (Kim-Muller et al., 2014; Martins et al., 2016; Mandai et al., 2018). FOXO4 is a suppressor in cancers. Nevertheless, recent studies have shown that FOXO4 is also a tumor promoter (Li et al., 2016; Choi et al., 2020; Wang et al., 2021); for example, the upregulated FOXO4 could inhibit the invasive and metastatic characteristics of cholangiocarcinoma cells by regulating focal adhesion kinase and F-actin dynamics. FOXO4 upregulation was in response to chemotherapeutic treatment in B-cell lymphoma, which is associated with a poor prognosis (Lee et al., 2009; Ryu et al., 2017; Jiang et al., 2018). FOXO4 is a factor regulating the cellular homeostasis of cancer cells, rather than just a tumor suppressor (Hornsveld et al., 2018). So far, there are some studies on the function of FOXO4 in CRC (Bian et al., 2018; Zhu et al., 2020; Bai et al., 2021). However, the accurate mechanism of FOXO4 and its regulation on APC2 are still poorly understood in CRC.

This study found that FOXO4 was downregulated in CRC tissues and positively correlated with APC2 and p(S37)-β-catenin. Additionally, FOXO4 reduced the migration and metastasis of CRC *via* the APC2/β-catenin axis, identified by *in vitro* and *in vivo* experiments. Our results suggested that FOXO4 might play an important role in tumor deterioration and is a potential treatment target in CRC.

## Materials and Methods

### Cell Culture and Transfection

Colorectal cancer cell lines (SW620, HCT116) and HEK293T cell lines were purchased from the American Type Culture Collection (Manassas, VA, United States). Cells were cultured in Dulbecco’s modified Eagle’s medium (DMEM, Gibco, Waltham, MA, United States) supplemented with 10% fetal bovine serum (FBS, Gibco) at 37°C in a humidified atmosphere containing 5% CO_2_.

The plasmid (FOXO4 overexpression or si-APC2) was transfected into cells by using EndoFectin Max transfection reagent (GeneCopoeia, Rockville, MD, United States) when the density of the cells reached about 70–80% in a six-well plate, 2 μg for one well. Incubated for about 4 h, the transfected medium was substituted by 2 ml of fresh complete DMEM. The siRNA sequences used were as follows: si-APC2-1 (5′-CAGATGGACATCACCAGCCTGTACA-3′/5′-TGTACAGGCT GGTGATGTCCATCTG-3′), si-APC2-2 (5′-GGGAACGGTG TTTCCTGCTGAATGA-3′/5′-TCATTCAGCAGGAAACACCG TTCCC-3′), and si-APC2-3 (5′-GGTCTTCTGGCTGTTGTC CATGTTG-3′/5′-CAACATGGACAACAGCCAGAAGACC-3′).

### Western Blot

The medium was removed and the cells were washed with phosphate-buffered saline (PBS). Cells were collected and lysed using a radioimmunoprecipitation assay lysis buffer (YEASEN, China) with 1/100 PMSF (YEASEN). The total protein of all samples was quantified using a bicinchoninic acid protein assay kit (YEASEN). Each sample containing an equal amount of total protein was separated by 10% SDS-PAGE. All subsequent operations conformed to the standard process. All primary antibodies in this paper were purchased from Abcam (England, United Kingdom). Antibodies used were as follows: anti-β-catenin (Santa Cruz, Dallas, TX, United States, sc7199, 1:1,000), anti-p (S37)-β-catenin (Abcam, ab47335, 1:1,000), anti-GSK3β (Abcam, ab131356, 1:1,000), anti-p (Y216)-GSK3β (CST, 9323s, 1:1,000), anti-vimentin (Proteintech, Chicago, IL, United States, 10366, 1:1,000), anti-E-cadherin (CST, Danvers, MA, United States, 3195, 1:1,000), anti-N-cadherin (CST, 13116, 1:1,000), anti-Snail (Abcam, ab180714, 1:1,000), anti-proliferating cell nuclear antigen (PCNA) (Santa Cruz, SC-25280, 1:1,000), anti-c-MYC (Abcam, ab32072, 1:1,000), anti-ERK1/2 (CST, CST-9102, 1:1,000), and anti-BCL2 (CST, CST-3498, 1:1,000).

### Quantitative Real-Time PCR

Total RNA was extracted using TRIzol reagent (Invitrogen, Waltham, MA, United States) and then quantified by NanoDrop (Thermo Fisher Scientific, Waltham, MA, United States). Next, RNA was reverse-transcribed to cDNA using the RevertAid RT Reverse Transcription Kit (Thermo Fisher Scientific). The mRNA expression was determined using SYBR Green qRT-PCR kit (Takara, China). Table 1 lists the primers used in this study. GAPDH was used as internal controls, and the relative expression level was calculated according to the 2^–ΔΔCt^ method.

**TABLE 1:** Primers used in this study.

**Primer ID**	**Primer sequences (5′–3′)**
APC2-F	GGCCAGGATGCCCGAAAATTA
APC2-R	AGCGAGCGGATGTGCTG
FOXO4-F	ATGGTCCGTACTGTACCCTACTTC
FOXO4-R	CTTTGCCGCTCTTGCCTC
ChIP-APC2-1-F	GACTTAGTTCCTCTTTGGGG
ChIP-APC2-1-R	CTGGGCATGTCTCTCTCTG
ChIP-APC2-2-F	AGTAGCTGGGACTACAGGCA
ChIP-APC2-2-R	GGATCACGAGGTCAGGAGAT
ChIP-APC2-3-F	AGAGTGAAACTCCATCTCAGAA
ChIP-APC2-3-R	CAAAGGGCTGGGATTACA
ChIP-APC2-NC-F	TGGCTGGGCATGGGAT
ChIP-APC2-NC-R	CAGGCTTGGGGTTGGG
GAPDH-F	GCAAGAGAGAGGCCCTCAG
GAPDH-R	TGTGAGGGAGATGCTCAGTG

### Immunohistochemistry

The tissues were sectioned into 5 μm, dewaxed, and rehydrated in sequence in xylene and alcohol. The sections soaked in ethylenediaminetetraacetic acid solution were heated to retrieve antigen at 100°C for 30 min, then cooled naturally at room temperature. Next, tissue sections were immersed in 0.3% hydrogen peroxide solution for 15 min, rinsed with PBS for 5 min, and blocked with 3% bovine serum albumin for 30 min. The tissue sections were incubated with the first antibodies for 20 h at 4°C and then with horseradish peroxidase-conjugated secondary antibody for 1 h, and diaminobenzene was used as the chromogen. Finally, the sections were dehydrated and mounted. The antibodies used were anti-FOXO4 (Thermo Fisher, 720154, 1:200) and anti-APC2 (Abcam, ab233753, 1:200).

As positive criteria, brown-yellow was considered positive. Two senior pathologists analyzed and evaluated the results according to the staining intensity and the percentage of stained cells. All results were determined by the unified scoring standard and double-blind method.

### Immunocytochemistry

Normal cells and cells transfected with plasmids for 24 h were fixed with 4% paraformaldehyde for 15 min and then perforated by 0.2% Triton X-100. Cells were incubated with the first antibodies for 20 h at 4°C and then with Alexa Fluor 594/488-conjugated secondary antibody for 1 h, and DAPI was used as the nuclear counterstain. All the above operations between different reagents were washed three times with PBS. An inverted fluorescence microscope photographed the typical areas. Antibodies used were as follows: anti-β-catenin (Santa Cruz, sc7199, 1:200), anti-CD133 (Abcam, ab276130, 1:200), and anti-ALDH1 (CST, 54135S, 1:200).

### Chromatin Immunoprecipitation

HEK293T (seeded in 10-cm dish) was overexpressed with FOXO4 for 48 h, the cell coverage rate was close to 100%, and then the chromatin immunoprecipitation (ChIP) assay process was performed according to the standard protocol.

### Wound-Healing Assay

Cells were seeded into six-well plates, about 10^6^ cells for one plate. When the cells completely covered the bottom of the plate, a straight line was evenly drawn using a 10-μl pipette tip. Then, the cells were cultured in DMEM with 1% FBS under standard culture conditions for 24 h. Healing was determined at four indicated times.

### Dual-Luciferase Activity Assay

293T cells were seeded in a 96-well culture plate. The corresponding plasmids were transfected when the cell coverage was about 70%. After 12 h of transfection, the fresh culture medium was changed and then cultured for 48 h. Cells were collected to detect luciferase activity according to the protocol of the manufacturer (Dual-Luciferase Reporter Gene Assay Kit, 11402ES60, YEASEN).

### Transwell Assay

The transwell test was conducted with a transwell chamber coated with Matrigel mix (BD Biosciences, Franklin Lakes, NJ, United States). A 100-μl 10^6^ cells/ml cell suspension was cultured in the upper chamber of the transwell cell for 24 h. The cell layer in the upper chamber was wiped, and cells in the lower chamber were fixed with 10% formaldehyde for 30 min and stained with 1% crystal violet. After washing with PBS two to three times, the cells were observed using an inverted microscope. Five fields were randomly selected for cell counting, and the average value was calculated.

### Construction of Stable HCT116 Cell Line by Lentivirus

About 50–100 HCT116 cells were thinly inoculated into a six-well plate; 12 h later, the lentivirus containing FOXO4 overexpression or si-APC2 fragment was added to the medium, respectively. Seventy-two hours later, the medium was replaced with fresh complete DMEM including puromycin (Sigma, Burlington, MA, United States) and then cultured for another 48 h. The cells were digested into a single-cell suspension and inoculated into a 96-well plate, with a maximum of one cell per well, and cultured in a complete medium with puromycin. The puromycin-resistant cells were authenticated as a stable cell line.

### Xenograft Tumor Models of Nude Mice

Our animal experiment was approved by the ethical committee of Guangzhou Forevergen Biosciences (Guangzhou, China, approval number: IACUC-G16043). Male BALB/c nude mice (3–4 weeks old) were randomly divided into eight groups (four groups for the spleen-injected groups and the other four for the liver-infiltrated groups); 5 × 10^6^ cells were injected into the spleen or liver, constructing two xenograft models. Then, 15 days (spleen-injected groups) or 20 days (liver-injected groups) later, mice were killed using the CO_2_ method, and the corresponding organs were removed to observe the tumors. In the process of the animal experiment, mice were also killed when the following conditions were met: (1) weight loss 20–25%, (2) total loss of appetite for 24 h, and (3) unable to stand up for 24 h without anesthesia or sedation.

### Statistical Analyses

Data analysis was conducted using GraphPad Prism v.7.0, presented as mean ± SEM. Statistical analysis was conducted by either Student’s *t*-test (two-group comparison) or one-way analysis of variance (more than two groups, Tukey’s multiple comparisons test was performed for difference analysis). The correlation between variables was analyzed by Spearman correlation coefficient. Asterisks represent ^∗^*P* < 0.05, ^∗∗^*P* < 0.01, ^∗∗∗^*P* < 0.001, and ^****^P < 0.0001.

## Results

### FOXO4 and APC2 Were Downregulated With a Positive Correlation in Colorectal Cancer

**FIGURE 1:**
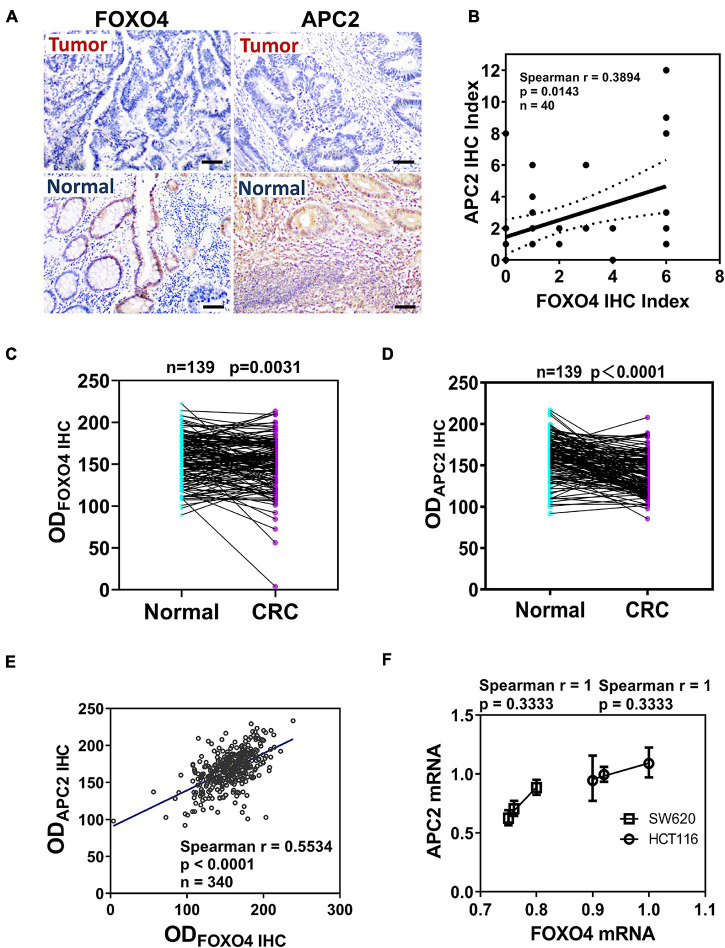
The expression and correlation of Forkhead box O4 (FOXO4) and adenomatous polyposis coli 2 (APC2) in patients with colorectal cancer. **(A)** The protein expression of FOXO4 and APC2 in colorectal cancer tissue and paracancerous tissue, detected by immunohistochemistry (IHC) (scale bar = 50 μm). **(B)** Spearman analysis shows the correlation between FOXO4 and APC2 analyzed by Spearman correlation coefficient. Two independent evaluations were performed for scoring the IHC index of FOXO4 and APC2 in CRC tissue of 40 patients, and its average value was used to analyze the correlation between FOXO4 and APC2 (*n* = 40, *P* = 0.0143). **(C,D)** IHC detected the expression of FOXO4 **(C)** and APC2 **(D)** in a tissue microarray (tumor tissue and paracancerous normal tissue) of 139 patients with CRC. Tissue microarray was scanned after IHC, and optical density was counted; a statistical test showed that the expression of FOXO4 and APC2 was higher in paracancerous normal tissue than in tumor tissue (*n* = 139). **(E)** The correlation between FOXO4 and APC2 in tissue microarray of 139 patients with CRC, presented by Spearman correlation coefficient (*n* = 340, *P* < 0.0001). **(F)** The correlation between *FOXO4* and *APC2* at the mRNA level in two colorectal cancer cell lines (HCT116 and SW620), analyzed by Spearman coefficient (*n* = 6, *P* = 0.3333).

To explore the correlation between FOXO4 and APC2 in CRC, we detected and compared the expression of FOXO4 and APC2 in normal tissues and tumors of patients with CRC by immunohistochemistry. Both FOXO4 and APC2 were expressed in normal tissues adjacent to carcinoma but less in tumor lesions (Figure 1A). The immunohistochemical results of FOXO4 and APC2 in tissues of 40 patients with CRC were evaluated, showing that FOXO4 was positively correlated with APC2 at the protein level in CRC (*P* = 0.0143) (Figure 1B). To ensure the stability of the above results, we used more CRC tissues to detect the expression of FOXO4 and APC2. The tissue microarray test results in 139 patients with CRC indicated that both FOXO4 and APC2 were downregulated significantly in CRC tumor lesions than normal tissue adjacent to carcinoma (Figures 1C,D). As mentioned, there was an apparent positive correlation between FOXO4 and APC2 in CRC tissues from 139 patients, analyzed by Spearman correlation coefficient (*P* < 0.0001) (Figure 1E). Meanwhile, the expressions of *FOXO4* and *APC2* were positively correlated at the mRNA level in the CRC cell line (Figure 1F). In short, both FOXO4 and APC2 were downregulated significantly in colorectal cancer compared with a standard control, with a positive correlation.

### FOXO4 Could Bind *APC2* and Regulate Its Expression

**FIGURE 2:**
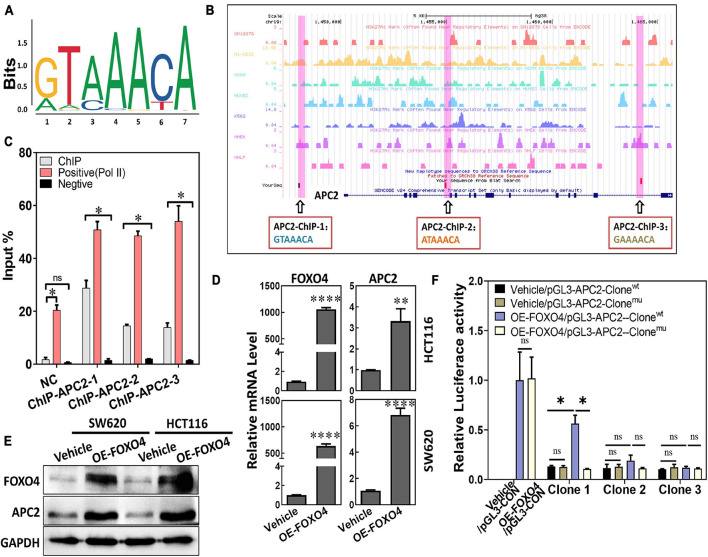
FOXO4 enhances the expression of APC2 as a transcription factor. **(A)** The motif sequences that FOXO4 can bind as a transcription factor, predicted by bioinformatics analysis. **(B)** The physical location and sequence of the APC2 promoter region, which was predicted as a possible motif sequence. **(C)** ChIP results showed that the FOXO4 protein combined the *APC2* gene sequence. FOXO4 protein was overexpressed in 293T cell lines and enriched by ChIP, and then three pairs of primers that targeted the *APC2* gene sequence were performed to test the binding of FOXO4 to the *APC2* sequence (*n* = 6 per group). **(D,E)** The expression of APC2 at the mRNA (*n* = 6 per group) **(D)** and protein **(E)** levels in HCT116 and SW620 cell lines at 48 h after FOXO4 overexpression. **(F)** The relative luciferase activity in 293T cell lines transfected with different vectors (*n* = 5 per group). *P < 0.05; **P < 0.01; ****P < 0.001.

As a transcription factor, the FOXO4 expression was closely positively correlated with APC2 in colorectal cancer, which suggested that FOXO4 may bind the *APC2* sequence and regulate its expression. “GTAAACA” was the possible sequence that FOXO4 could bind as a transcription factor, analyzed by bioinformatics (Figure 2A). Similarly, we predicted the physical location of the motif sequence in the promoter region of *APC2* and selected three potential motif sequences as follow-up verification sites, including “GTAAACA” (Figure 2B). FOXO4 was overexpressed in 293T cells and enriched by the ChIP method. The selected motif sequence of *APC2* was detected by qPCR, whose results showed that the input proportion of three *APC2* ChIP sequences was significantly higher than that of the negative control (Figure 2C; Supplementary Figure 1). Finally, we found APC2 was upregulated at the mRNA and protein levels in colorectal cancer cell lines (SW620 and HCT116) with FOXO4 overexpression (OE) for 48 h (Figures 2D,E), and these similar results were verified by luciferase assay (Figure 2F). In summary, FOXO4, as a transcription factor, could bind the motif sequence of *APC2* and enhance its expression in CRC cells.

### FOXO4 Promoted Degradation of β-Catenin Based on Phosphorylation and Inhibited the EMT Process in Colorectal Cancer Cell

**FIGURE 3:**
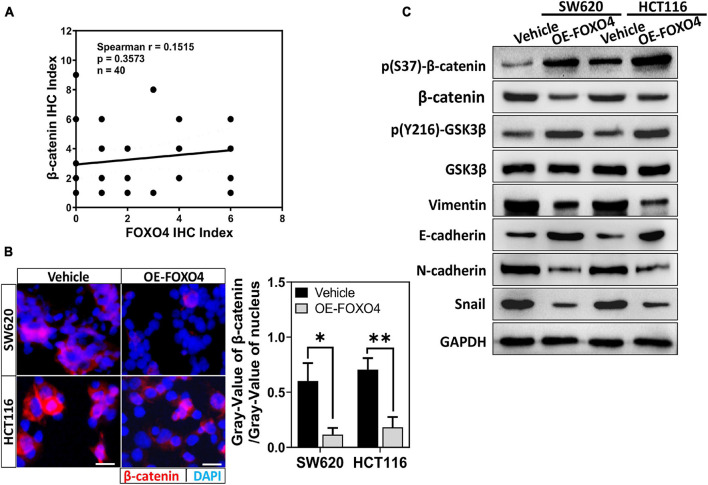
FOXO4 promoting β-catenin phosphorylated degradation and inhibiting the EMT process. **(A)** The correlation between FOXO4 and p(S37)-β-catenin in CRC tissue, shown by Spearman analysis. Two independent evaluations were performed for scoring the IHC index of FOXO4 and p(S37)-β-catenin in CRC tissue of 40 patients, and its average value was used to analyze the correlation between FOXO4 and p(S37)-β-catenin (*n* = 40, *P* = 0.3573). **(B)** Immunocytochemistry results of β-catenin in HCT116 and SW620 cell lines at 48 h after FOXO4 overexpression (scale bar = 10 μm). **(C)** The protein expression of β-catenin, p(S37)-β-catenin, and EMT-related proteins in HCT116 and SW620 cell lines at 48 h after FOXO4 overexpression, detected by Western blot. *P < 0.05; **P < 0.01.

Adenomatous polyposis coli 2 is a tumor-suppressor gene in the colon. Its typical action is to promote the migration of β-catenin from the nucleus and its phosphorylation. According to the above results, we explore whether FOXO4 regulates β-catenin. The protein expression of FOXO4 and p(S37)-β-catenin was detected in CRC tissues from 40 patients by immunohistochemistry (IHC), and their IHC index was analyzed for correlation. The Spearman correlation coefficient explained that p(S37)-β-catenin was positively correlated with FOXO4 (Figure 3A). β-Catenin protein was reduced in both nucleus and the whole cell when FOXO4 was overexpressed in SW620 and HCT116 cell lines (Figure 3B and Supplementary Figure 2). By further verification, with FOXO4 overexpression in SW620 and HCT116, the molecules of mesenchymal transformation (vimentin, N-cadherin, and Snail) were downregulated. However, the molecules of epithelial transformation (E-cadherin) were upregulated; p(Y216)-GSK3β was slightly upregulated, but the change of GSK3β was not noticeable; p(S37)-β-catenin was upregulated, and β-catenin was not significantly changed (Figure 3C). Together, FOXO4 was positively correlated with p(S37)-β-catenin in CRC tissue. Meanwhile, FOXO4 could promote degradation of β-catenin based on phosphorylation to inhibit the EMT process in the colorectal cancer cell.

### FOXO4 Inhibited the Stemness of Colorectal Cancer Cell

**FIGURE 4:**
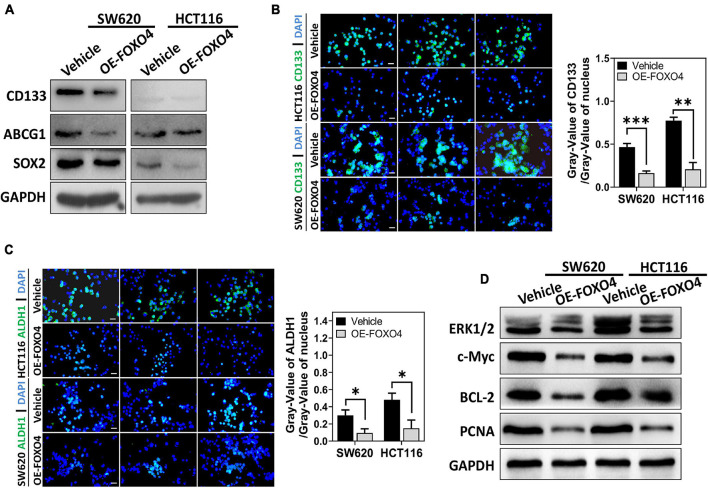
FOXO4 overexpression inhibiting the stemness of colorectal cancer cells. **(A)** The expression of stemness-related proteins (CD133, ABCG1, and SOX2) at the protein level in HCT116 and SW620 cell lines, tested by Western blot at 48 h after FOXO4 overexpression. **(B,C)** Immunocytochemistry showed the CD133 **(B)** and ALDH1 **(C)** protein in CRC cells (scale bar = 10 μm). **(D)** The expression of ERK1/2, c-Myc, BCL-2, and PCNA at the protein level in CRC cells, after FOXO4 overexpression. *P < 0.05; **P < 0.01; ***P < 0.001.

SW620 and HCT116 were overexpressed with FOXO4, and the proteins involved in cell stemness (CD133, ABCG1, SOX2) were detected. Western blot results indicated that CD133, ABCG1, and SOX2 were downregulated caused by FOXO4 overexpression (Figure 4A). Similarly, immunocytochemical analysis showed that both CD133 and ALDH1 were decreased in CRC cell lines with FOXO4 overexpression compared with the standard control (Figures 4B,C). The protein levels of ERK1/2, c-Myc, BCL-2, and PCNA were significantly reduced in both SW620 and HCT116 treated with FOXO4 overexpression (Figure 4D). In a word, the stemness of CRC cells could be inhibited by FOXO4 overexpression.

### FOXO4 Inhibited EMT, Migration, and Metastasis of Colorectal Cancer Cells *via* APC2

**FIGURE 5:**
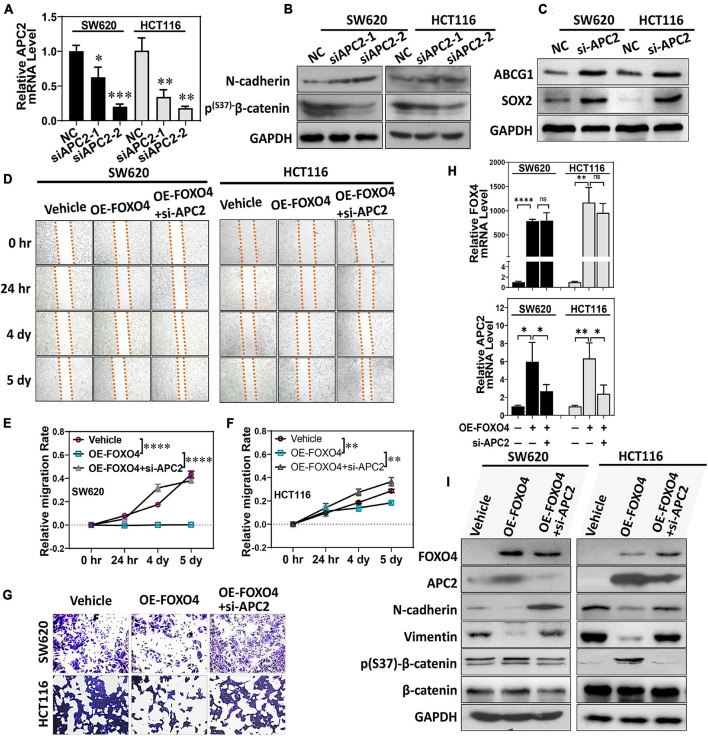
FOXO4 regulating APC2 to inhibit EMT and migration of colorectal cancer cells. **(A)** qPCR was used to detect the interference efficiency of two siRNAs targeting the *APC2* gene (*n* = 6). **(B,C)** SW620 and HCT116 were knockdowns by siRNAs targeting the APC2 for 48 h, and the expression of N-cadherin, p(S37)-β-catenin **(B)**, and ABCG1 and SOX2 was detected by Western blot. **(D)** Evaluating the effect of FOXO4 overexpression and APC2 knockdown for SW620 and HCT116 cell lines on cell migration capacity by wound-healing assay. SW620 and HCT116 cells with FOXO4 overexpression or si-APC2 were wounded and incubated. Healing was determined at four indicated times. **(E,F)** Comparison of cell mobility of three groups in **(C)** (*n* = 6). Six scratches in each group were selected and photographs were taken at the indicated times, and then cell mobility was calculated and compared. **(G)** Transwell assay for cell migration. Colorectal cancer cell lines HCT8 and HCT116 were transfected with FOXO4 overexpression or APC2 knockdown. **(H)** The relative mRNA level of FOXO4 and APC2 in SW620 and HCT116 cell lines treated with FOXO4 overexpression and APC2 knockdown, respectively. **(I)** Protein expression in SW620 and HCT116 cell lines was performed by different interventions. *P < 0.05; **P < 0.01; ***P < 0.001; ****P < 0.001.

We designed two siRNA sequences that targeted *APC2* and tested their interference efficiency. Both siRNAs could significantly reduce the expression of APC2 in colorectal cancer cell lines. Among these, siAPC2-2 was more efficient (Figure 5A). At the same time, N-cadherin was upregulated, and p(S37)-β-catenin was downregulated (Figure 5B). Both ABCG1 and SOX2 were increased in SW620 and HCT116 treated with APC2 knockdown (Figure 5C; Daly et al., 2017; García-Prat et al., 2020). We observed the effects of FOXO4 overexpression and APC2 knockdown on the migration of colorectal cancer cells by wound-healing assays (Figure 5D). Statistical results at four specified times displayed that OE-FOXO4 groups closed wounds more tardily than control, but the OE-FOXO4 + si-APC2 groups were close to or even faster than control in both SW620 and HCT116 (Figures 5E,F). To further test this, three groups (standard, OE-FOXO4, OE-FOXO4 + si-APC2) of SW620 and HCT116 were conducted for the transwell assay. FOXO4 OE inhibited the migration of both SW620 and HCT116 obviously, which would be relieved by APC2 knockdown (Figures 5G,H). Additionally, APC2 knockdown significantly restored the downregulation of N-cadherin and vimentin induced by FOXO4 OE (Figure 5I).

**FIGURE 6:**
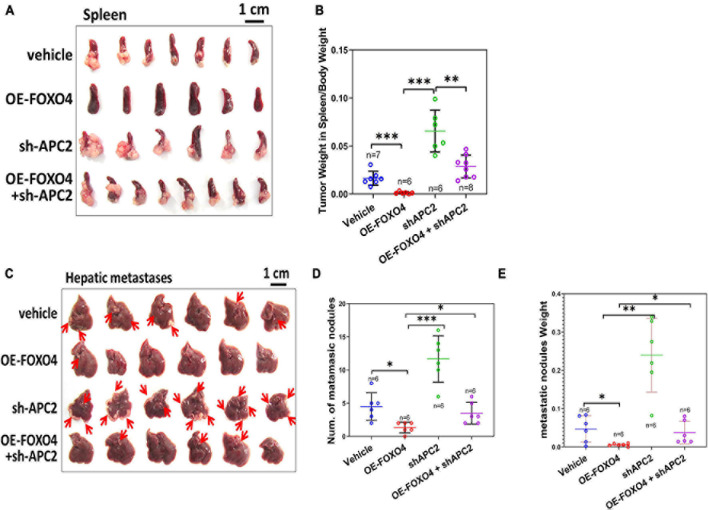
FOXO4 inhibiting the proliferation of colorectal cancer cells *via* APC2 *in vivo*. **(A)** The resected spleens to detect metastasis of HCT116 from four groups of xenograft nude mice on the 15th day. Stable HCT116 cell line was constructed *via* lentivirus for FOXO4 overexpression or APC2 knockdown. About 5 × 10^5^ cells were injected into the spleen of nude mice; the spleens were taken for observation after 15 days (vehicle: *n* = 7, OE-FOXO4: *n* = 6, sh-APC2: *n* = 6, OE-FOXO4 + sh-APC2: *n* = 8). **(B)** The weight ratio of tumor to spleen in the four groups was monitored on the 15th day. **(C)** The resected livers to detect metastasis of HCT116 from the four groups of nude mice on the 20th day; 5 × 10^5^ cells from the four groups were injected into the liver of xenograft nude mice and incubated for 20 days (*n* = 6). **(D,E)** The number **(D)** and weight ratio **(E)** of tumor to liver in the four groups were monitored on the 20th day (*n* = 6). *P < 0.05; **P < 0.01; ***P < 0.001.

To further verify the role of FOXO4 and APC2 in tumor invasion and metastasis *in vivo*, we injected four groups of SW620 cells (normal, OE-FOXO4, sh-APC2, OE-FOXO4 + sh-APC2) into the spleen and liver of nude mice, respectively. Fifteen days after injection, the spleens were dissected for observation. There was no apparent tumor load in the OE-FOXO4 group, and the size of tumor tissues in sh-APC2, OE-FOXO4 + sh-APC2, and standard groups was arranged from large to small (Figure 6A). Statistical analysis confirmed the above results (Figure 6B). Similarly, few tumor tissues were observed in the liver of the OE-FOXO4 group, but the sh-APC2 group had the most tumor tissues, followed by the standard and sh-APC2 groups (Figure 6C and Supplementary Figure 3). The statistical analysis of the numbers and weight of liver tumor tissues was consistent with the results of the naked-eye observation (Figures 6D,E). To summarize, FOXO4 could regulate a series of related genes to inhibit EMT, migration, and metastasis *in vivo* of CRC cells *via* APC2.

## Discussion

In this research, compared with the standard, FOXO4, APC2, and p(S37)-β-catenin were downregulated in CRC tumors, with a positive correlation. Bioinformatic analysis predicted that FOXO4 might bind the *APC2* gene sequence, which was confirmed by ChIP. Overexpressed FOXO4 has vital solid biological functions in CRC cell lines, including enhancing the expression of APC2, promoting the phosphorylation of β-catenin and inhibiting the EMT process, and weakening the stemness of CRC cells. In the end, overexpression of FOXO4 significantly reduced the migration and *in vivo* metastasis of CRC cells, which could be reversed by siRNA-mediated APC2 knockdown.

Forkhead box O4 is a transcriptional factor involving multiple physiological functions and regarded as a tumor suppressor in many cancers (Hornsveld et al., 2018). Previous studies have shown that non-coding RNA (circRNA or microRNA) could regulate the proliferation, migration, and metastasis of CRC cells by targeting FOXO4 (Ryu et al., 2017; Hornsveld et al., 2018), which indicated that FOXO4 plays a vital role in the pathological process of CRC. In this study, FOXO4 was downregulated in CRC tissues compared with paracancerous tissue, consistent with previous studies.

However, FOXO4 plays the role of tumor suppressor only by regulating the expression of downstream effector genes. It is essential to explore the specific regulatory pathway of FOXO4 in CRC. APC2 can suppress the Wnt/β-catenin pathway strongly by promoting phosphorylated degradation of β-catenin, considered the most potential CRC deterioration regulator. The methylation of APC2 was upregulated obviously in CRC, suggesting the occurrence of CRC (Liu et al., 2011; Bian et al., 2018; He et al., 2018). The Wnt/β-catenin pathway was activated to promote EMT by inhibiting the expression of APC2 in CRC (Novellasdemunt et al., 2015; Cheng et al., 2019; Meng et al., 2020). APC2 and p(S37)-β-catenin were downregulated in CRC tumors, with a positive correlation with FOXO4, which suggested that FOXO4 functions by regulating APC2 and the Wnt/β-catenin pathway. The ChIP results showed that FOXO4 could bind the motif of *APC2* and enhance its expression, which indicated that FOXO4 is a key transcriptional regulation factor of APC2, and FOXO4, improving the APC2/β-catenin axis, would be the mechanism of FOXO4 inhibiting CRC. *In vivo* and *in vitro* experiments showed that FOXO4 inhibited EMT, migration, and *in vivo* metastasis of colorectal cancer cells by regulating the APC2/β-catenin axis.

There were some significant achievements in this current study, accompanied by some limitations at the same time. As a powerful transcription factor, FOXO4 can regulate the expression of many genes. This article focused only on the APC2/β-catenin axis, rather than other promising genes regulated by FOXO4 and which played a role in CRC disease progression. Previous studies have shown that most of the mutated *APC* genes in colorectal tumors lack β-catenin-binding regions and fail to inhibit Wnt signaling, leading to overproliferation of tumor cells (Senda et al., 2005). We found that the inhibitory effect of FOXO4 on the migration and *in vivo* metastasis of CRC cells can be reversed by APC2 knockdown. For patients with CRC and inactivation or decreased activity of *APC2* gene mutation, the treatment strategy targeting FOXO4 may be an invalid choice, so mutation screening of *APC* genes may be a necessary prestep in CRC treatment by targeting FOXO4. More research will be carried out in the future, including all the above deficiencies.

## Conclusion

In summary, we demonstrated that FOXO4 has a tumor-suppressor role that could inhibit EMT, migration, and *in vivo* metastasis in colorectal cancer by regulating the APC2/β-catenin axis, which revealed the function and mechanism of FOXO4 in colorectal cancer, providing a potential therapeutic strategy for patients with CRC.

## Data Availability Statement

The original contributions presented in the study are included in the article/Supplementary Material, further inquiries can be directed to the corresponding author.

## Ethics Statement

The animal study was reviewed and approved by the Ethics Committee of Guangzhou Forevergen Biosciences (Guangzhou, China, approval number: IACUC-G16043).

## Author Contributions

YS conceived and designed the study and critically revised the manuscript. YS and LW performed the experiments, analyzed the data, and drafted the manuscript. XX, PH, JW, and XT participated in the study design, study implementation, and manuscript revision. ML contributed to response to the comments of reviewers and provided the funding support. All authors read and approved the final manuscript.

## Conflict of Interest

The authors declare that the research was conducted in the absence of any commercial or financial relationships that could be construed as a potential conflict of interest.

## Publisher’s Note

All claims expressed in this article are solely those of the authors and do not necessarily represent those of their affiliated organizations, or those of the publisher, the editors and the reviewers. Any product that may be evaluated in this article, or claim that may be made by its manufacturer, is not guaranteed or endorsed by the publisher.

## Supplementary Material

Supplementary Figure 1 Chromosomal localization and sequence of 3 fragments of APC2 gene. Those APC2 gene fragments were predicted to bind to FOXO4 transcription factors.

Supplementary Figure 2 Immunocytochemistry results of β-catenin in HCT116 and SW620 cell lines at 48 h after FOXO4 overexpression (scale bar = 10 μm).

Supplementary Figure 3 Hematoxylin and eosin staining of livers detecting metastasis of HCT116 from four groups of nude mice on 20th day.
