# Supplementary figures and images for: FOXO4 Inhibits the Migration and Metastasis of Colorectal Cancer by Regulating the APC2/β-Catenin Axis
Source: Front Cell Dev Biol. 2021 Sep 23;9:659731. doi: 10.3389/fcell.2021.659731 (PMC8495124; doi:10.3389/fcell.2021.659731)

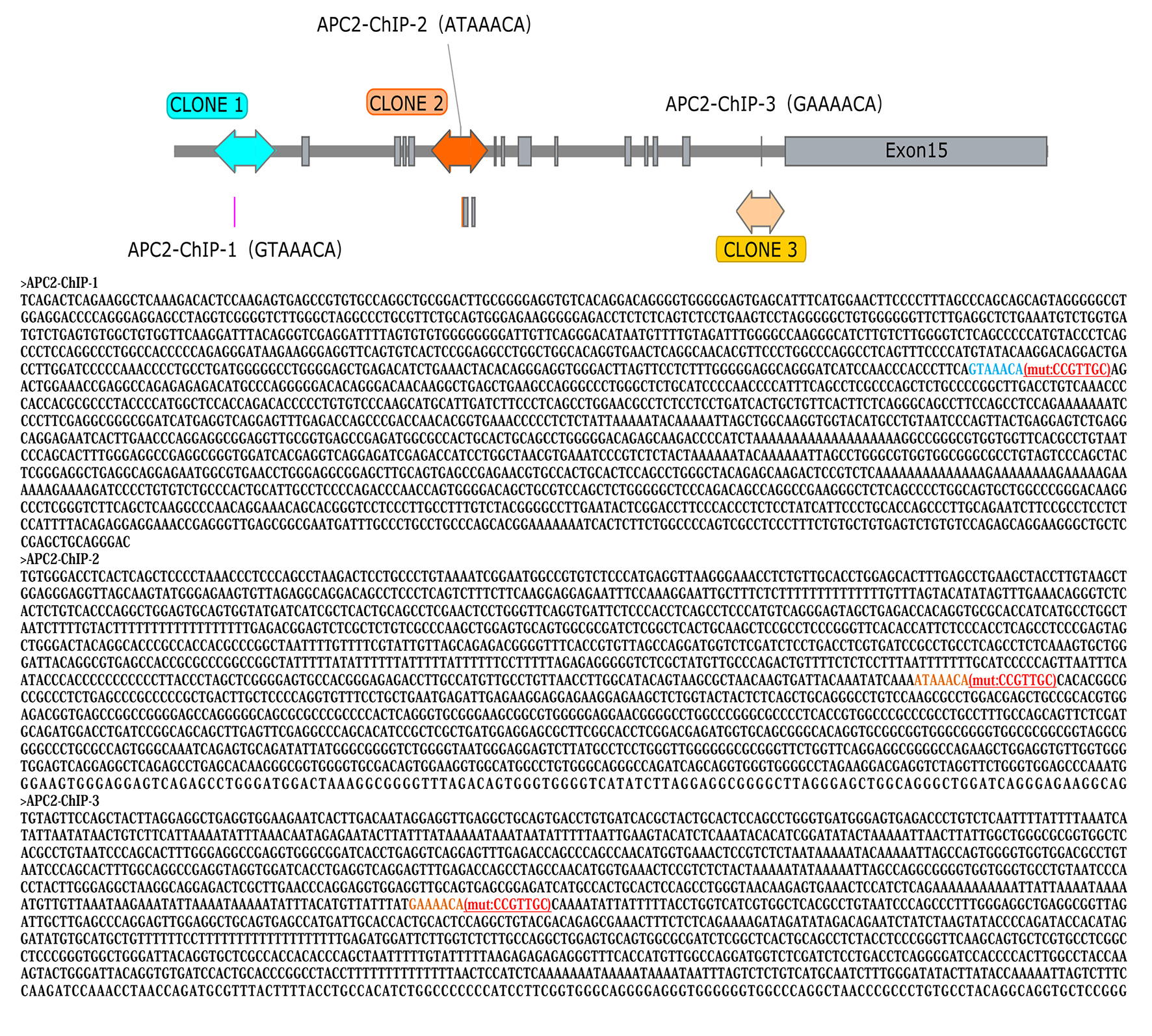

Supplement: Supplementary Figure 1 — Chromosomal localization and sequence of 3 fragments of APC2 gene. Those APC2 gene fragments were predicted to bind to FOXO4 transcription factors. [file Image_1.TIF]

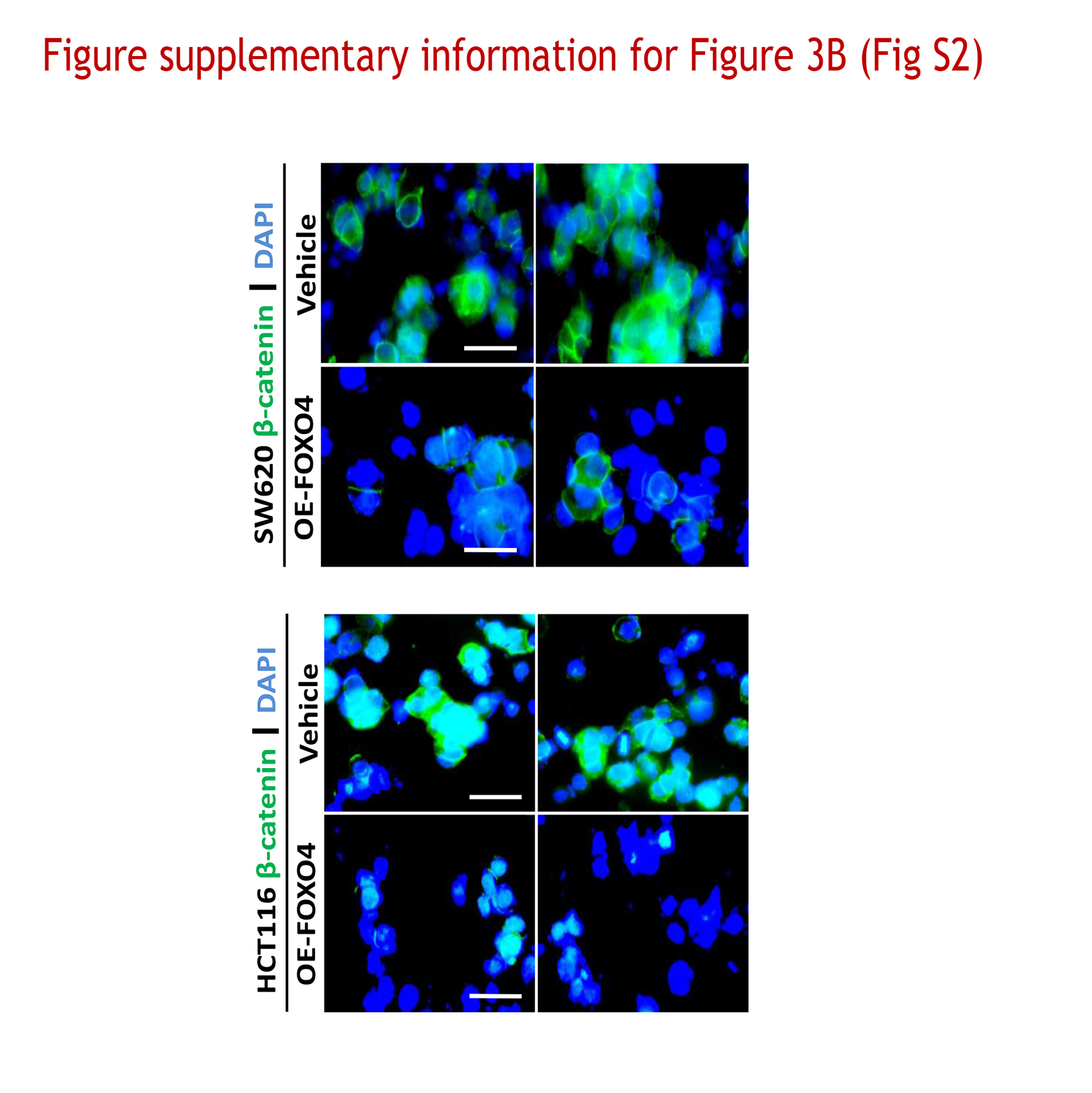

Supplement: Supplementary Figure 2 — Immunocytochemistry results of β-catenin in HCT116 and SW620 cell lines at 48 h after FOXO4 overexpression (scale bar = 10 μm). [file Image_2.TIF]

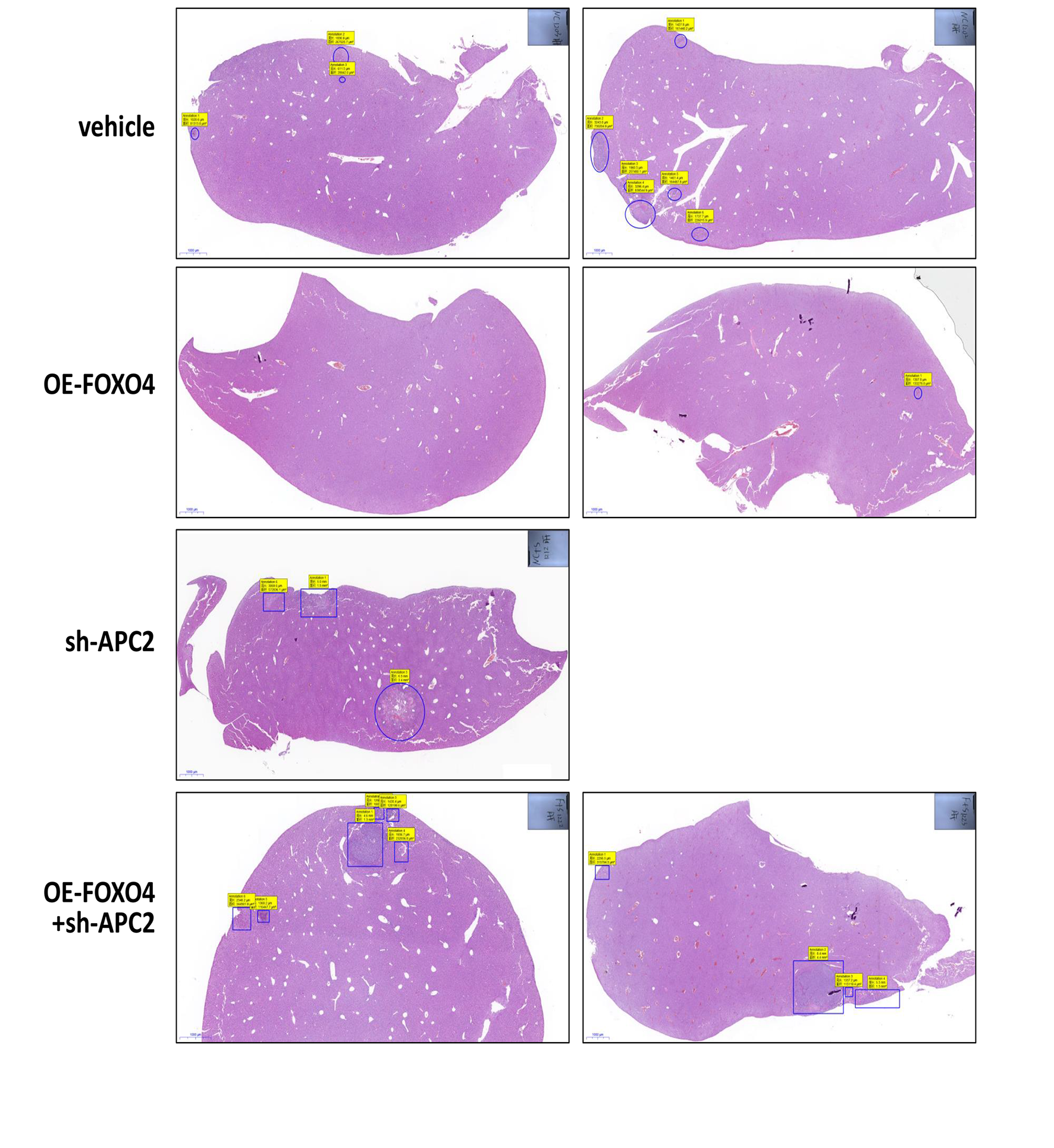

Supplement: Supplementary Figure 3 — Hematoxylin and eosin staining of livers detecting metastasis of HCT116 from four groups of nude mice on 20th day. [file Image_3.TIF]
